# Application of a free preputial tube graft coupled with urethral plate urethroplasty combined with a Buck's fascia integral covering for the single-stage repair of severe hypospadias

**DOI:** 10.3389/fsurg.2022.1047104

**Published:** 2023-01-06

**Authors:** Wei Zhou, Changpei Li, Fan Xia, Qi Zhang, Yanxing Chen

**Affiliations:** Department of Urology, The Children's Hospital of Xiamen, Xiamen, China

**Keywords:** severe hypospadias, Buck's fascia, free preputial tube graft, urethral plate, surgical treatment

## Abstract

**Objective:**

To assess the outcome of a free preputial tube graft coupled with urethral plate urethroplasty combined with a Buck's fascia integral covering (BFIC) for the single-stage repair of severe hypospadias in children.

**Materials and methods:**

A retrospective study was performed on 40 children with hypospadias who were treated in our hospital from December 2017 to February 2022. The inclusion criteria were as follows: (1) the patient had proximal hypospadias, or penile curvature over 30° after degloving the penis; and (2) the patient underwent surgery for hypospadias for the first time. A free preputial tube graft coupled with urethral plate urethroplasty combined with a Buck's fascia integral covering was performed, and all patients were followed up for more than 6 months.

**Results:**

After degloving the foreskin, the urethral orifice was retracted to the perineum and scrotum in 20 cases, penoscrotal in 8 cases, and penile in 12 cases. Single-stage repair was achieved without complications in 34 (85%) patients. The remaining six patients experienced postoperative complications: urethrocutaneous fistula occurred in five cases and glans dehiscence with urethrocutaneous fistula in one case. No urethral diverticulum occurred in any case. A neomeatus with a vertically oriented slit-like appearance was achieved at the tip of the glans in all cases, with one exception.

**Conclusion:**

The single-stage operation with a free preputial tube graft coupled with urethral plate urethroplasty combined with a Buck's fascia integral covering in the treatment of severe hypospadias achieves favorable functional and cosmetic outcomes.

## Introduction

The repair of hypospadias with obvious penile curvature, especially proximal hypospadias, has always been a difficult problem for pediatric urologists. The choice of method is largely dependent on the relative subjective assessment of the patient's abnormal anatomy (including urethral orifice location, urethral plate quality, and degree of chordee) and the surgeon's experience and preference (1, 2). In proximal hypospadias, the urethral plate is usually hypoplastic and has severe penile curvature that requires dissection of the urethral plate. One-stage or staged surgery using a vascularized prepuce or preputial graft is currently the main approach (3, 4). There have been few reports about free preputial tube grafts; moreover, the reported results of surgery vary, and the complication rate can be as high as 20%–68% (5, 6). To our knowledge, the use of a Buck’s fascia covering in hypospadias surgery was first reported in 2017 (6). There have been reports of applying the Buck's fascia as integral covering tissues, providing an intermediate layer to cover the neourethra in tubularized incised plate (TIP) and similar procedures in China from 2019, and the overall results showed that the effect was better than that of pedicled dartos fascia (7–9).

Since December 2017, we have used a free preputial tube graft coupled with urethral plate urethroplasty combined with a Buck's fascia integral covering (BFIC) for the single-stage repair of severe hypospadias and have achieved good results. The report is as follows.

## Patients and methods

This study was conducted in the Department of Urology at our hospital between December 2017 and February 2022. The inclusion criteria were as follows: (1) the patient had proximal hypospadias (according to the Hadidi (10) classification); (2) the curvature of the penis after degloving was 30° or more; and (3) the patient underwent surgery for hypospadias for the first time. The exclusion criteria were as follows: (1) reoperation; and (2) small glans (maximum diameter of glans <9 mm). All operations were performed by the same experienced surgeon. The age range of the 40 patients was 6 months to 11 years (mean age 33.8 ± 31.9 months), and the curvature of penis was in the range of 30°–135° after degloving (mean 63.6° ± 24°).

## Surgical procedure

**Figure 1:**
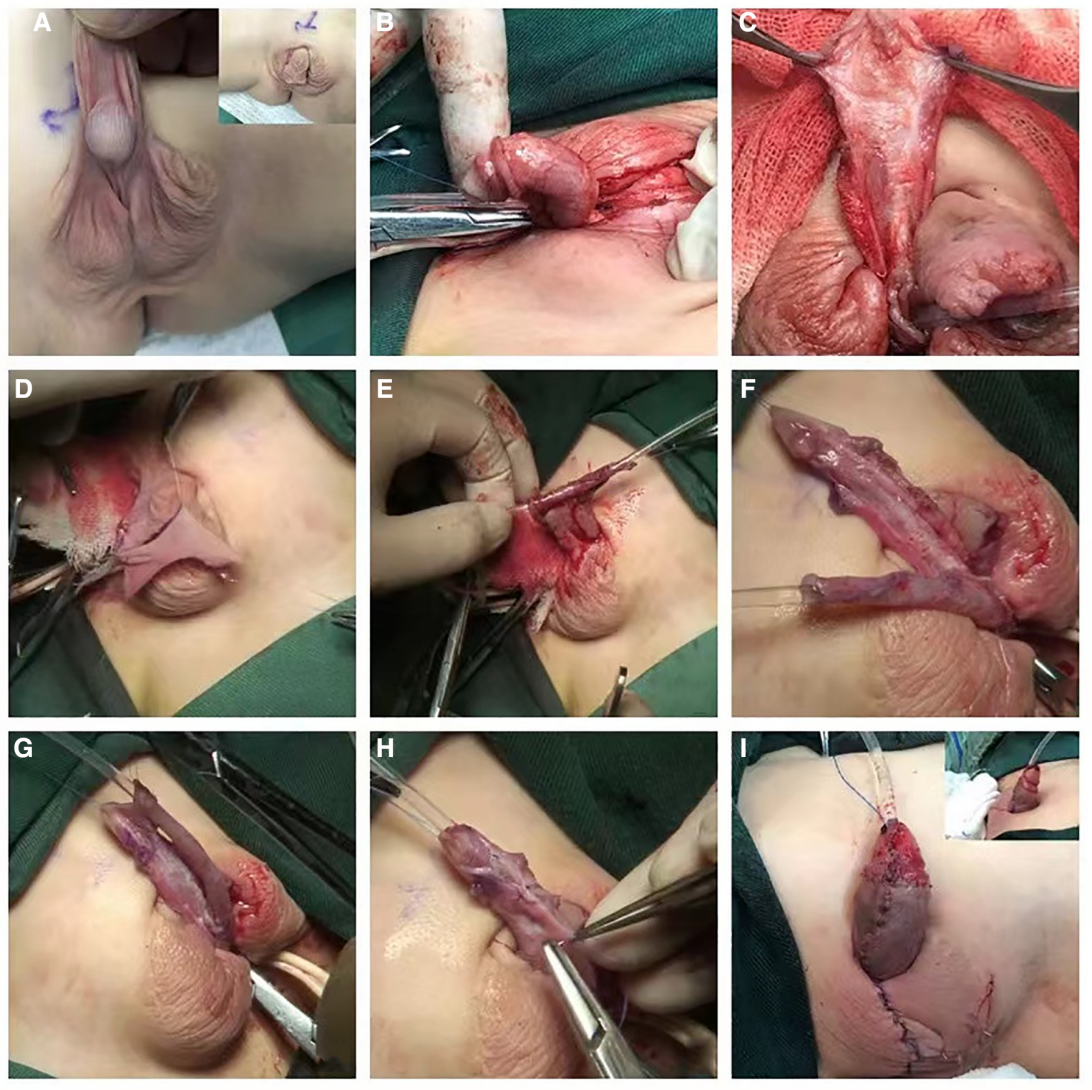
(**A**) Pre-surgery appearance. (**B,C**) The prepuce was completely degloved. Buck's fascia on both sides of the urethral plate was carefully separated, the ventral urethral plate was incised transversely in the middle and separated to two parts. (**D–F**) A fusiform flap which was longer than required was dissected on the dorsal prepuce. The middle part was rolled and sutured to a tube shape, then the vascular pedicle of the flap was excised, (**G,H**) the resulting free graft was anastomosed to the separated urethra plate with a interrupted suture, the BF and the glans wings were sutured as an intermediate layer to wrap the neourethra. (**I**) Post-surgery appearance.

**Figure 2:**
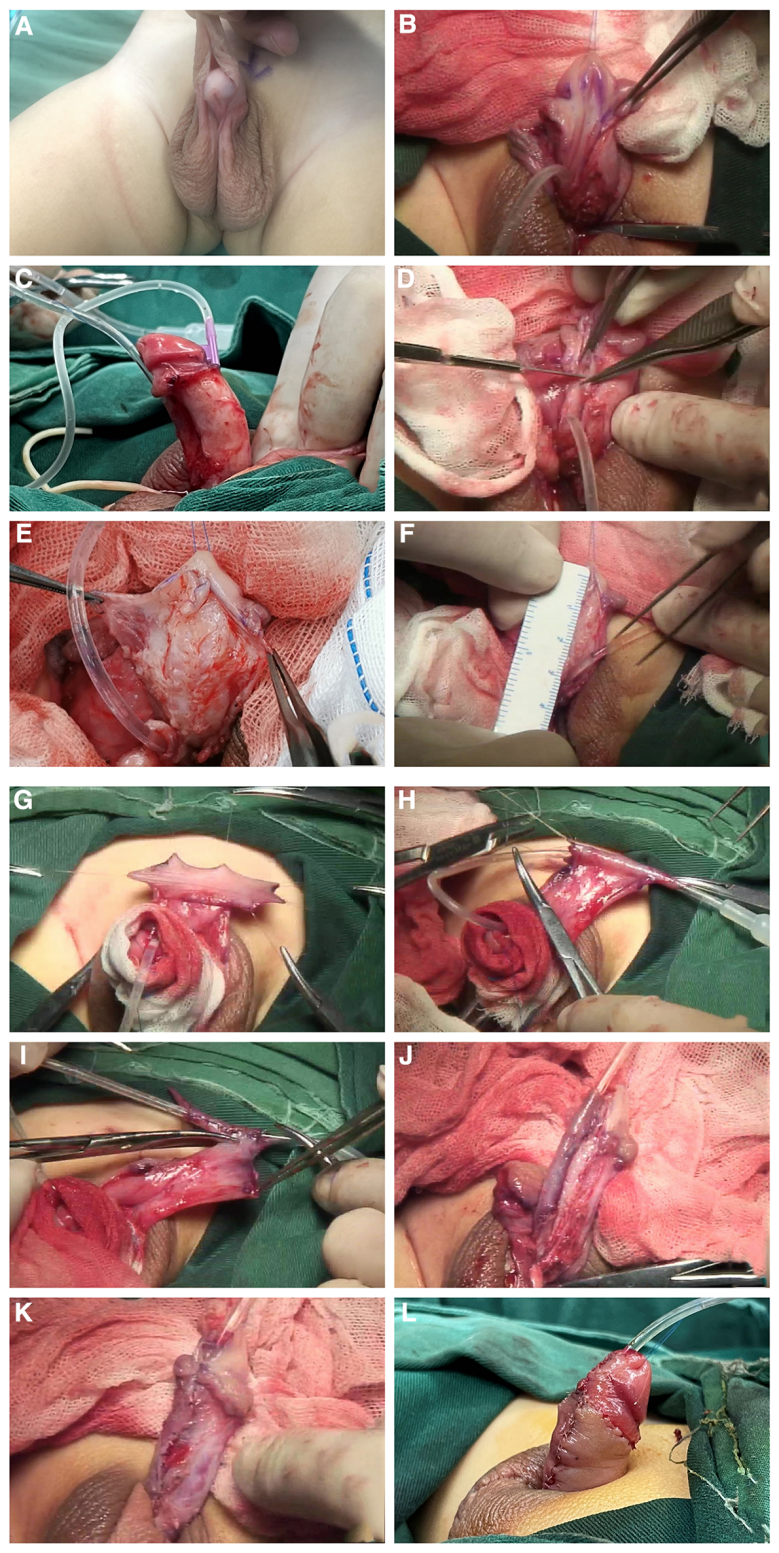
(**A**) Pre-surgery appearance. (**B–E**) The prepuce was completely degloved. Buck's fascia on both sides of the urethral plate was carefully separated, the ventral urethral plate was incised transversely in the middle and separated to two parts. (**F–I**) A fusiform flap which was longer than required was dissected on the dorsal prepuce. the middle part was rolled and sutured to a tube shape, then the vascular pedicle of the flap was excised. (**J**) The resulting free graft was anastomosed to the separated urethra plate with interrupted sutures. (**K**) The Buck's fascia together with the divergent spongiosum and the glans wings were sutured at the midline to completely cover the neo urethra. (**L**) Post-surgery appearance.

We sutured the traction thread on the dorsal side of the glans; a longitudinal parallel incision was made along both sides of the urethral plate, and the proximal end surrounded the urethral orifice in a “U” shape. At approximately 0.3 cm from the coronal sulcus, the dorsal foreskin was circumcised and completely degloved. We then performed an artificial erection test; if the penis was curved over 30°, we used a knife to carefully separate the Buck’s fascia on both sides of the urethral plate to the 3 and 9 o'clock positions (Figures 1B,C and 2E).

**Figure 3:**
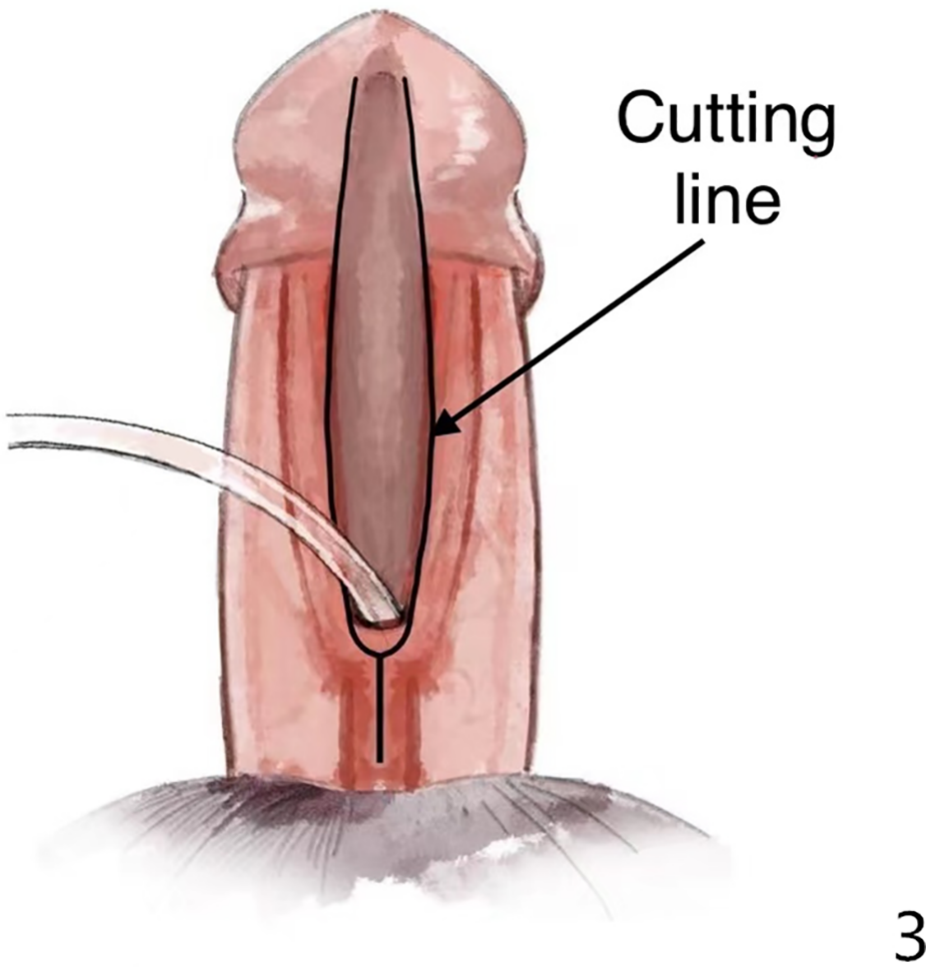
We separated Buck's fascia and glans wings along the black line in the figure. The divergent spongiosum lying on Buck's fascia was preserved. The urethral corpus cavernosum and Buck's fascia were separated together.

We widely separated the Buck's fascia along both sides of the urethral plate on the ventral side of the penis, and the divergent urethral spongiosum lying on Buck's fascia was preserved. The distal end was incised along the edge of the urethral plate to the top of the glans, separated with the two wings of the glans cavernous body. The proximal end extended to the normal urethral spongiosa below the urethral orifice (Figure 3). The urethral corpus cavernosum and Buck's fascia were separated together so that the neourethra could be firmly covered later. When separating the Buck's fascia, it is necessary to start at the distal and proximal ends of the most curved portion and then gradually separate the most curved portion to avoid damaging the Buck's fascia here.

**Figure 4:**
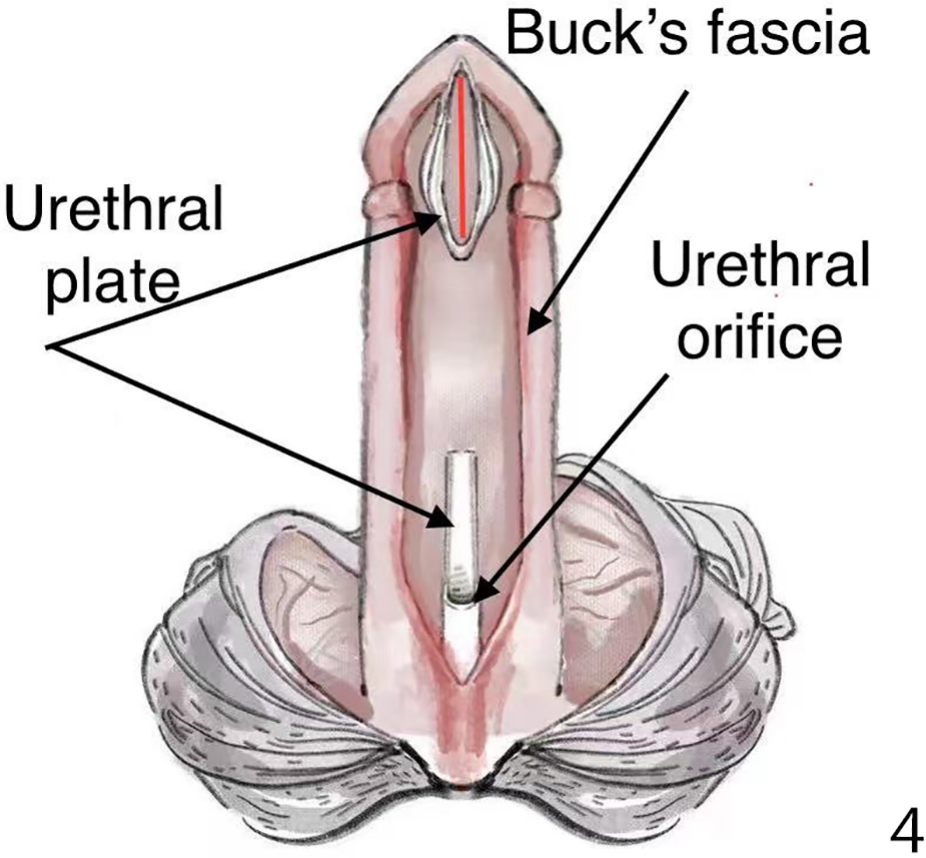
The ventral urethral plate was incised transversely in the middle and separated to two parts, and the distal part of the urethral plate was deeply incised longitudinally (red line in Figure 4).

During the separation of the Buck's fascia, the urethral plate was transected from the middle, which corrected the curvature and facilitated the separation of the Buck's fascia at the same time. We always incised the distal urethral plate longitudinally to gain better space in the glans (red line in Figure 4).

We reserved the distal plate as the combination of onlay and tip, and the proximal plate as the combination of onlay. We sutured the free prepuce graft tube to the distal and proximal plates obliquely “onlay” (Figure 6).

**Figure 5:**
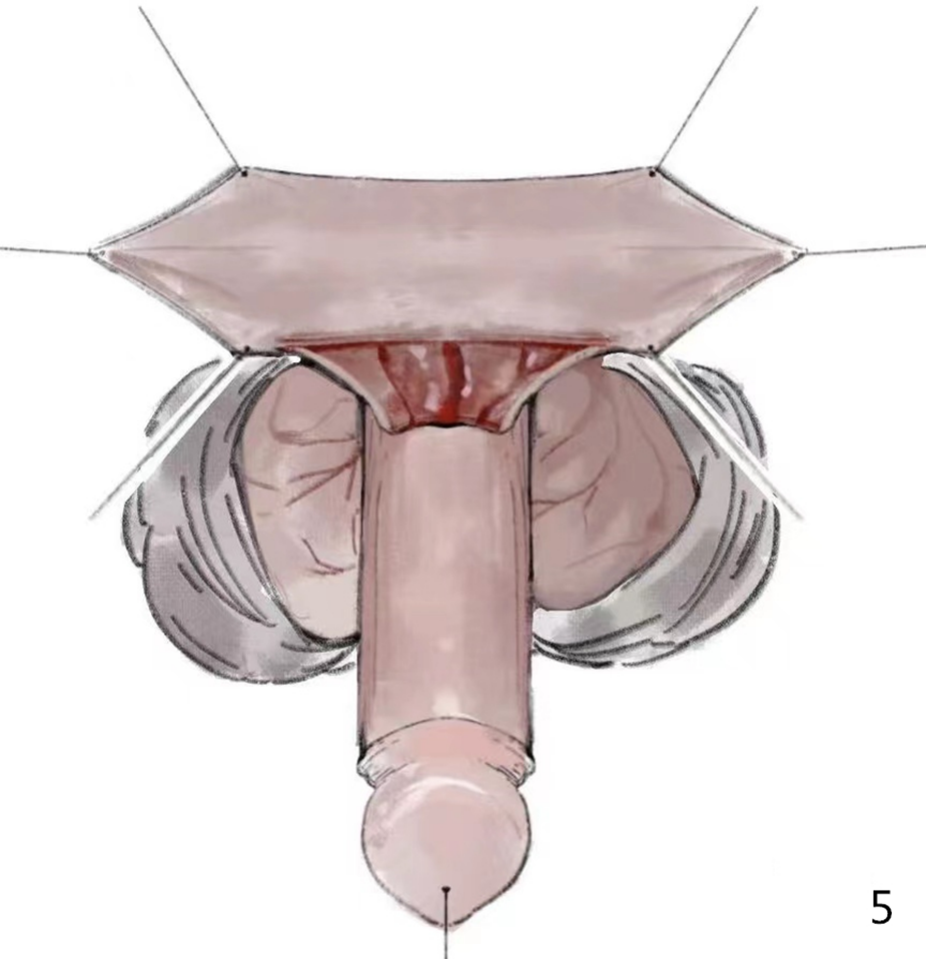
A fusiform flap which was longer than required was dissected on the dorsal prepuce.

We then measured the distance between the urethral orifice and the glans under traction to determine the expected maximum length of the free prepuce. The missing urethral plate was filled with the length of the tubular free foreskin. A fusiform flap that was longer than required was dissected on the dorsal prepuce (Figure 5). The width of the prepuce flap is generally 15 mm (range 12–20 mm). Around the urinary catheter (No. 8 balloon catheter), the middle flap was rolled and sutured to a tube shape, then the vascular pedicle of the flap was excised, after which the resulting free tube graft was anastomosed to the separated urethra plate with interrupted 6–0 MONOCRYL sutures. The medial part of the glanular tissue was appropriately trimmed so that the glans accommodated the No. 8 catheter loosely and without tension.

**Figure 6:**
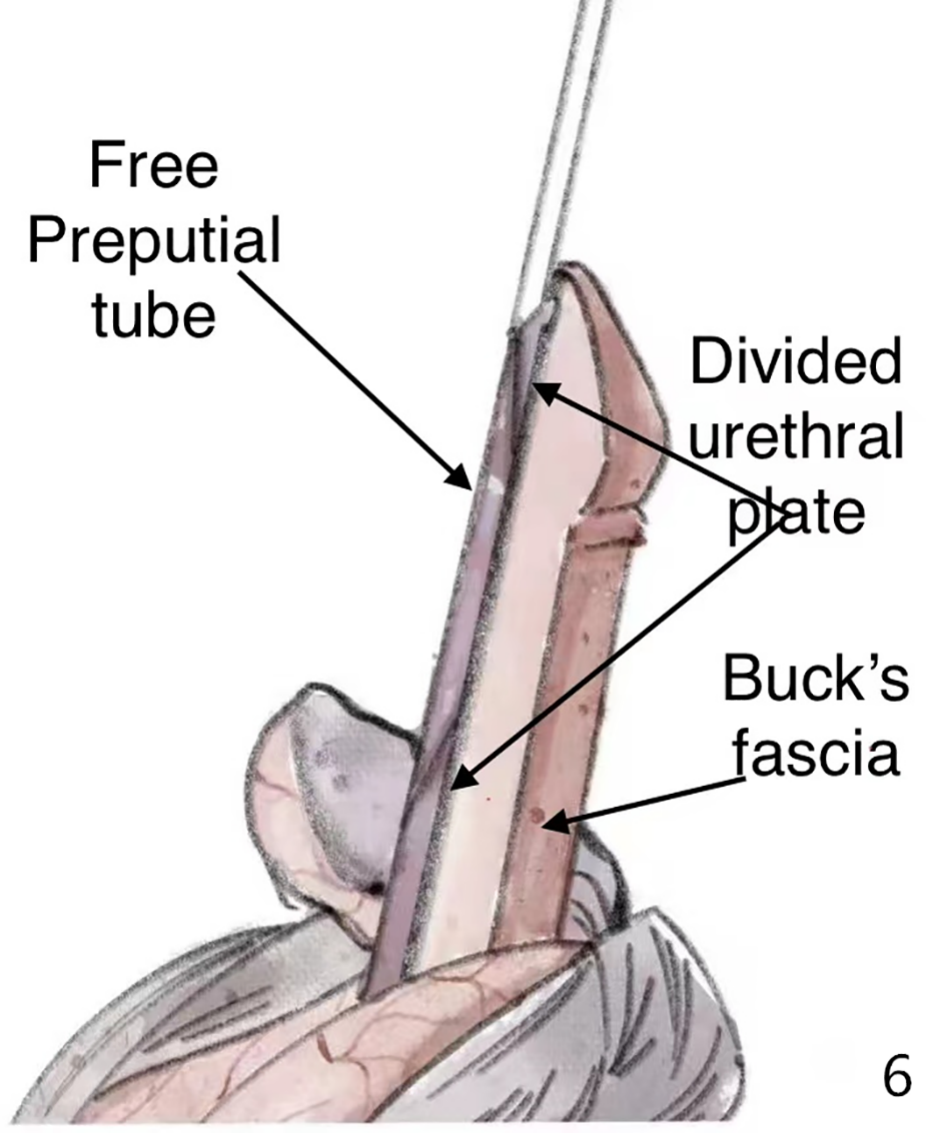
The resulting free graft was anastomosed to the separated urethra plate with interrupted sutures.

The distal end of the divergent spongiosum lying on the Buck's fascia was attached to the glans tissue and its proximal end was continued with the normal urethral cavernous body. The Buck's fascia, together with the spongiosum and the glans wings, were then sutured at the midline to completely cover the neourethra (Figures 2J,K).

In the process, the ventral Buck's fascia of the penis was relieved, its original curvature was straightened, the Buck's fascia was also extended after the contralateral suture, and a Z-shaped suture could be used to further lengthen the ventral Buck's fascia under conditions. The high-grade curvature was then moderately relieved in the erection test.

We made Baskin-type dorsal midline plications to completely correct the ventral curves in all cases, except for two without dorsal folds.

Skin closure was achieved using Byars’ flaps.

Other abnormalities, such as penile-scrotal transposition, scrotal split, and bilateral or unilateral cryptorchidism, were all repaired at the same time.

The bandage was removed 5–7 days after the operation, and the urinary catheter was removed 2–3 weeks after the operation, with routine oral antibiotics during that period.

## Postoperative follow-up

The patients had a regular follow-up in our outpatient clinic at 4 weeks, 3 months, and 6 months after surgery. After that, they visited us freely if they had any anxiety. The follow-up time ranged from 6 months to 2 years 11 months, with a mean of 1 year 7 months.

The contents of the evaluation included: urethral stricture, urinary fistula, glans dehiscence, recurrence of penile curvature, and urethral diverticulum. A urethral stricture was defined as symptomatic strictures (dysuria), or the urethral orifice was too narrow to insert an 8-Fr catheter, or the urinary flow rate curve was a plateau obstructive curve. Urethral diverticulum was defined as a balloon-like expansion of the skin of the neourethra during voiding, and a urethral fistula was defined as a urethral-cutaneous fistula requiring surgical intervention. Glans dehiscence was defined as the retraction of the urethral orifice into the coronal sulcus. The recurrence of penile curvature was defined as a postoperative curvature over 30°.

## Results

A total of 40 patients with hypospadias were included in the present study, with an age range of 6 months to 11 years, including three cases of disorders of sex development (DSD) with chromosomal abnormalities, two cases of bilateral cryptorchidism, and one case of unilateral cryptorchidism. The maximum width of the glans was 9–13 mm (mean width 10.9 ± 1.13 mm). After correcting the chordee, the urethral orifice was retracted to the perineum and scrotum in 20 cases, penoscrotal in 8 cases, and penile in 12 cases.

Postoperative complications consisted of a urethral fistula in six cases. All were healed by reoperation and were located at the proximal urethra anastomosis. No cases had a urethral stricture or urethral diverticulum, and one case of glans dehiscence did not affect urination and was not treated. There was no recurrence of penile curvature at follow-up.

## Discussion

Hypospadias with significant penile curvature, especially proximal hypospadias, is still a challenge for many pediatric urologists because of the intractable and complicated management of the transected urethral plate. For single-stage or staged reconstruction, various techniques have been described. Although the use of vascularized local penile or preputial skin is the mainstay of hypospadias repair nowadays, complications such as urethral stricture, urinary fistula, diverticulum, and torsion of the penis are also prone to occur (1, 3, 11). Free graft reconstruction of the neourethra is generally used in staged techniques, such as preputial or buccal mucosal grafts, which are currently commonly used (12).

Since the initial article on the use of a free preputial graft for hypospadias surgery by Devine and Horton, the results have varied (13). With the increasing number of articles on the application of free preputial grafts in single-stage urethroplasty, there is also controversy about the application effects (8, 14–16).

Hendren et al. (17) performed free preputial graft urethroplasty in 103 children with proximal hypospadias, under proximal urethrostomy; there was a lower complication rate of 8.9%. It is believed that the free preputial graft, like various other pedicled flap techniques, can provide a satisfactory functional and cosmetic outcome. Stock et al. (18) also came to a similar conclusion that grafts and pedicled flaps had the same success rate, following a total of 77 patients who underwent single-stage preputial free graft urethroplasty; 84% were successful. Recently, Japanese scholars Obara et al. (19) retrospectively evaluated 56 patients with free preputial tube graft urethroplasty; 14 cases developed postoperative complications and they proposed that this method was an ideal method for repairing hypospadias with severe penile curvature.

Many scholars believe that free grafts are only suitable for staged surgery. An inherent problem with the single-stage graft urethroplasty is the increased likelihood of urethral orifice and proximal anastomotic strictures. The subsequent fusion and revascularization of the graft is unpredictable in the early postoperative period, and circumferential and longitudinal scar atrophy of the neourethra may occur, leading to complications such as urethral/orifice stricture and recurrence of the penile curvature. The staged surgery overcomes these problems: in the first stage of surgery, the graft can be accurately and appropriately obtained, it can be completely sutured to the corpus cavernosum, and the pressure bandage can be further fixed to promote its revascularization and fusion. In the second stage, the graft has fully stabilized to its final size, allowing for the precise planning of the caliber of the neourethra and urethral orifice (4, 5, 15). However, some clinical practices have not confirmed the abovementioned presumed advantages of staged surgery: scar contractures sometimes require regrafting during secondary surgery, and complication rates can be unsatisfactory (13, 20).

When free grafts are used, it is important to cover the neourethra with a well-vascularized tissue as an intermediate layer to reduce complications such as urethrocutaneous fistulas. At present, the dartos fascia, scrotal fascia, divergent spongiosum, and tunica vaginalis flap are commonly used as covering layers. However, there is no accepted conclusion as to which one is better.

Since the use of a Buck's fascia covering in surgery for hypospadias was first reported in 2017 (6), there have been reports of applying the Buck's fascia as integral covering tissues, providing an intermediate layer to cover the neourethra in TIP and similar procedures in China from 2019. The overall results showed that the effect was better than that of pedicled dartos fascia (7–9)

The Buck's fascia is a layer of tissue close to the tunica albuginea. It extends from the Scarpa's fascia in the abdomen to the penis and surrounds the penile cavernosum and corpus spongiosum. It has a high tensile strength and toughness comparable to the rectus abdominis sheath that closes the abdominal wall (21). In a prospective controlled study by Baba et al. (6), 160 patients with middle and distal hypospadias were included and were followed up for 2 years; the Buck's fascia significantly reduced the rate of postoperative urethral fistula (2.5% in the Buck's fascia group, and 12.5% in the dartos fascia group). Recently, Faqihi et al. (22) retrospectively analyzed the data of 50 patients with mid-distal hypospadias who underwent TIP surgery and found that the complication rate of the Buck's fascia integral covering was 6%, which could effectively reduce the risk of postoperative urinary fistula and glans dehiscence. Zhang et al. (9) reported 384 cases of free preputial tube graft urethroplasty with the BFIC technique; the postoperative complication rate of these proximal hypospadias was 9.9%. With the application of the BFIC technique, excellent results have been achieved in middle and distal hypospadias and severe hypospadias (6–9, 16).

The Buck's fascia covers the neourethra, and together with the cavernous body, provides good anatomical support to overcome the aforementioned shortcomings.

In addition, the division of the corpus spongiosum lying on the Buck's fascia is located at the proximal end of the meatus and extends laterally up to the glans in a fan-shaped position. Although the divergent corpus spongiosum is weaker in the proximal group than in the distal group (23), it may provide a reliable vascular bed along with the penile corpus cavernosum to maximize the survival of the free preputial tube graft.

The Buck's fascia is obtained in situ with little anatomical dissection. After the reconstruction of the anatomy of the Buck's fascia, other abnormalities, such as penile-scrotal transposition, scrotal split, and bilateral or unilateral cryptorchidism, can be easily treated at the same time, and the best cosmetic result can be achieved.

The Buck's fascia is a more suitable, natural, and firmer layer than other tissues, avoiding complications of urethral diverticulum and reducing the risk that other tissues may cause, such as the dartos flap and the tunica vaginali flaps that may bring penis torsion and swelling. After the anatomy of the Buck’s fascia is reconstructed, the penoscrotal transposition and the bifid scrotum, which are common in severe hypospadias, can be easily treated at the same time without any issues. There is no need to have palliative or staged surgery for fear of damaging the blood supply of the neourethra. The Buck's fascia is expected to be the most ideal covering material in hypospadias repair surgery.

In the study by Chen et al., Zhang et al., and Obara et al. (8, 9, 19), the free preputial tube was sutured directly to the top of the glans, which may be prone to cause stenosis of the anastomosis. Obara et al. also reported the risk of prolapse of the urethral orifice when the graft tube was longer than required. In this operation, we combined TIP and onlay techniques in the glans urethroplasty. We believe that it can avoid stenosis of the distal urethra as much as possible. At the same time, it can ensure the slit-like appearance of the urethral orifice just like TIP.

Obara et al. (19) were worried about the long-term recurrence of penile curvature after surgery and avoided ensured that the length of the free tube did not exceed 35 mm. Chen et al. and Zhang et al. (8, 9) reported the use of a tube graft combined with the proximal Duplay. In our group, the longest preputial flap reached 50 mm; as the urethral plate was used for coupling, the longest rolled-tube flap only needed to be 30 mm. Coupling the urethral plate to shape the urethra allows us to require relatively shorter free preputial tissue, and has certain advantages in children with insufficient inner preputial materials.

In our postoperative follow-ups, we have not seen any recurrence of penile curvature exceeding 30°. A long-term follow-up may be required, especially in adolescence.

This report is a single-center study with a small sample size and a short follow-up time. Further multicenter studies, a larger sample size, and long-term follow-up are needed to obtain more reliable results.

## Data Availability

The original contributions presented in the study are included in the article/**Supplementary Material**, further inquiries can be directed to the corresponding author.

## References

[1] Snodgrass W Bush N. Primary hypospadias repair techniques: a review of the evidence. Urol Ann. (2016) 8(4):403–8. 10.4103/0974-7796.192097 28057982 PMC5100143

[2] Springer A Tekgul S Subramaniam R. An update of current practice in hypospadias surgery. Eur Urol Suppl. (2017) 16(1):8–16. 10.1016/j.eursup.2016.09.006

[3] Gong EM Cheng EY. Current challenges with proximal hypospadias: we have a long way to go. J Pediatr Urol. (2017) 13(5):457–67. 10.1016/j.jpurol.2017.03.024 28549608

[4] Babu R Chandrasekharam V. Meta-analysis comparing the outcomes of single stage (foreskin pedicled tube) versus two stage (foreskin free graft & foreskin pedicled flap) repair for proximal hypospadias in the last decade. J Pediatr Urol. (2021) 17:681–9. 10.1016/j.jpurol.2021.05.014 34099397

[5] Castagnetti M El-Ghoneimi A. Surgical management of primary severe hypospadias in children: systematic 20-year review. J Urol. (2010) 184(4):1469–75. 10.1016/j.juro.2010.06.044 20727541

[6] Baba AA Wani SA Bhat NA Buck's fascia repair with glanuloplasty in hypospadias surgery: a simple approach with excellent outcome. J Pediatr Urol. (2017) 13(6):633.e1–e5. 10.1016/j.jpurol.2017.06.015 28789936

[7] Lin S He SH Xu HH Li LZ Xu D. Application of Buck's fascia for restoring complete wrapping of neourethra with corpus spongiosum for hypospadias during tubularized incised plate urethroplasty. Chin J Pediatr Surg. (2020) 41(08):733–8. 10.3760/cma.j.cn421158-20190606-00390

[8] Wu YL Chen HS Xu YB Wu YL Hu Y. Clinical analysis of severe hypospadias repair with free inner prepuce tube. J. Clin. Urol. (2020) 35(6):431–4. 10.13201/j.issn.1001-1420.2020.06.003

[9] Zhang Y Chao M Zhang W-P Tang Y-M Chen H-C Zhang K-P Using buck's fascia as an integral covering in urethroplasty to restore the anatomical structure of the penis in one-stage hypospadias repair: a multicenter Chinese study comprising 1,386 surgeries. Front Pediatr. (2021) 9:695912. 10.3389/fped.2021.695912 34434906 PMC8380957

[10] Chan YY Bury MI Yura EM Hofer MD Cheng EY Sharma AK. The current state of tissue engineering in the management of hypospadias. Nat Rev Urology. (2020) 17(3):162–75. 10.1038/s41585-020-0281-4 32024995

[11] Wang YP Chen F Xie H. Surgical advances for proximal hypospadias. J Clin Ped Sur. (2018) 17(11):866–71. 10.3969/j.issn.1671-6353.2018.11.015.

[12] Snodgrass W Bush N. Staged tubularized autograft repair for primary proximal hypospadias with 30-degree or greater ventral curvature. J Urol. (2017) 198(3):680–6. 10.1016/j.juro.2017.04.019 28400187

[13] Faure A Bouty A Nyo YL O'Brien M Heloury Y. Two-stage graft urethroplasty for proximal and complicated hypospadias in children: a retrospective study. J Pediatr Urol. (2016) 12(5):286.e1–e7. 10.1016/j.jpurol.2016.02.014 27020542

[14] Powell CR Mcaleer I Alagiri M Kaplan GW. Comparison of flaps versus grafts in proximal hypospadias surgery. J Urol. (2000) 163(4):1286–8; discussion 1288–89. 10.1016/S0022-5347(05)67762-2 10737530

[15] Fine R Reda EF Zelkovic P Gitlin J Freyle J Franco I Tunneled buccal mucosa tube grafts for repair of proximal hypospadias. J Urol. (2015) 193(5 Suppl):1813–7. 10.1016/j.juro.2014.10.093 25817150

[16] Tang YM Wang XJ Mao Y Chen SJ Liu M Chen YJ. Short term effectiveness of hypospadias repair with free inner prepuce tube. Chin J Reparative Reconstructive Surg. (2016) 30(07):866–70. 10.7507/1002-1892.20160176 29786324

[17] Hendren WH Horton CE Jr. Experience with 1-stage repair of hypospadias and chordee using free graft of prepuce. J Urol. (1988) 140(5 Pt 2):1259–64. 10.1016/S0022-5347(17)42019-2 3054165

[18] Stock JA Cortez J Scherz HC Kaplan GW. The management of proximal hypospadias using a 1-stage hypospadias repair with a preputial free graft for neourethral construction and a preputial pedicle flap for ventral skin coverage. J Urol. (1994) 152(6 Pt 2):2335–7. 10.1016/S0022-5347(17)31671-3 7966736

[19] Obara K Hoshii T Hoshino S Yamana K Anraku T Maruyama R Free tube graft urethroplasty for repair of hypospadias. Urol Int. (2020) 104(5–6):386–90. 10.1159/000504146 31801150

[20] Chan YY D'Oro A Yerkes EB Rosoklija I Balmert LC Lindgren BW Challenging proximal hypospadias repairs: an evolution of technique for two stage repairs. J Pediatr Urol. (2021) 17(2):225.e1–225.e8. 10.1016/j.jpurol.2020.12.008 33388263

[21] Cunha GR Sinclair A Risbridger G Hutson J Baskin LS. Current understanding of hypospadias: relevance of animal models. Nat Rev Urology. (2015) 12(5):271–80. 10.1038/nrurol.2015.57 25850792

[22] Fahad F Abdulrahman A Mohammed A Daood A Waleed A Demetrios JK Outcome of Buck's fascia repair with wingless glanuloplasty in distal penile hypospadias. Afr J Urol. (2021) 21(1):506. 10.1186/s13063-020-04454-4

[23] Bao X Huang Y Lyu Y Xi Z Xie H Fu Q A histomorphological study of the divergent corpus Spongiosum surrounding the urethral platein hypospadias. Urology. (2020) 144:188–93. 10.1016/j.urology.2020.04.139 32707270

